# Nobiletin ameliorates hepatic ischemia and reperfusion injury through the activation of SIRT-1/FOXO3a-mediated autophagy and mitochondrial biogenesis

**DOI:** 10.1038/s12276-019-0245-z

**Published:** 2019-04-26

**Authors:** Theodomir Dusabimana, So Ra Kim, Hye Jung Kim, Sang Won Park, Hwajin Kim

**Affiliations:** 1 Department of Pharmacology, Institute of Health Sciences, Gyeongsang National University School of Medicine, Jinju, 52727 Republic of Korea; 2 Department of Convergence Medical Sciences, Institute of Health Sciences, Gyeongsang National University Graduate School, Jinju, 52727 Republic of Korea

**Keywords:** Hepatotoxicity, Acute inflammation

## Abstract

Hepatic ischemia and reperfusion injury are characterized by impaired autophagy, mitochondrial dysfunction, and subsequent compromise of cellular homeostasis following hepatic surgery or transplantation. Nobiletin, a natural flavonoid, is a beneficial antioxidant that possesses anti-inflammatory and anti-cancer activities. We investigated the effect of nobiletin on hepatic IR injury and described the underlying mechanisms. C57BL/6 mice were subjected to 60 min of partial hepatic ischemia, treated with nobiletin (5 mg/kg) or vehicle at the start of reperfusion, and killed at 5 h of reperfusion. Hepatic ischemia and reperfusion increased hepatocellular oxidative damage, inflammation, and cell death, but these changes were alleviated upon nobiletin treatment. Nobiletin increased the expression of proteins that control autophagy, mitochondrial dynamics, and biogenesis. Specifically, the SIRT-1/FOXO3a and PGC-1α pathways were activated by nobiletin. IR-induced AKT activation was associated with FOXO3a phosphorylation, which resulted in a significant reduction in the nuclear FOXO3a levels and potentially attenuated autophagy-regulatory gene expression. Nobiletin increased FOXO3a expression and its nuclear translocation via the inhibition of AKT. Specific inhibition of SIRT-1 abolished the protective effect of nobiletin, causing decreased FOXO3a expression, followed by autophagy induction and decreased PGC-1α expression and mitochondrial dynamics. Taken together, our data indicate that SIRT-1 directly mediates the protective effect of nobiletin against hepatic ischemia and reperfusion injury. The activation of autophagy and mitochondrial function through the SIRT-1/FOXO3a and PGC-1α pathways indicate that nobiletin could have therapeutic potential for treating hepatic ischemia and reperfusion injury.

## Liver injury: Citrus peel to the rescue

Nobiletin, an antioxidant found in citrus peel, may protect the liver from reperfusion injury, damage following blood flow interruption. When blood flow is restricted and then restored, as in transplant, surgery, or shock, cells are injured, largely due to damage to the cellular powerhouses, the mitochondria. Nobiletin is known to have many benefits, including anti-cancer and anti-inflammatory activities, but its mechanism of action is not well understood. Sang Won Park and Hwajin Kim, at the Gyeongsang National University School of Medicine, in Jinju, South Korea, and co-workers, investigated how nobiletin might protect the liver against interruption of blood flow. They found that nobiletin triggered cells to dismantle damaged mitochondria and produce new, functioning mitochondria, greatly reducing liver damage. These results illuminate how nobiletin works and may lead to better treatments for liver reperfusion injury.

## Introduction

Hepatic ischemia and reperfusion (IR) injury, caused by blood deprivation followed by reperfusion, occurs in various clinical settings, including hepatic resection surgery, transplantation, and shock. The pathophysiology of hepatic IR injury contributes to an increased rate of acute liver failure, graft rejection, and chronic hepatic dysfunction^1,2^. Hepatic IR aggravates hepatic tissue damage through ATP depletion, production of reactive oxygen species (ROS), and inflammatory responses, which cause necrotic and apoptotic cell death^3^. IR injury affects parenchymal hepatocytes, nonparenchymal cells (liver sinusoidal endothelial, Kupffer, and hepatic stellate cells), and extrahepatic components (cytokines)^4^. Unfortunately, effective therapeutic strategies for treating or preventing this devastating syndrome are clinically limited, despite our advanced understanding of IR injury mechanisms.

Autophagy is a highly conservative cellular process that degrades and recycles misfolded or dysfunctional proteins and damaged organelles to maintain cellular homeostasis^5^. However, in cases of alcoholic liver, non-alcoholic fatty liver, viral hepatitis, toxin-induced hepatitis, and hepatocellular carcinoma, autophagy is impaired^6^. Autophagy induction ameliorates hepatic IR dysfunction by activating the heme oxygenase-1 (HO-1) pathway^7,8^.

Mitochondria are major organelles responsible for ATP generation and cellular homeostasis. During the ischemic period, ATP is depleted due to a lack of oxygen, resulting in a switch to anaerobic respiration. After reperfusion, mitochondrial function is disrupted, leading to excessive ROS generation, the opening of mitochondrial permeability transition pores, inflammation, and subsequent cell death^3,9^. Hepatic IR injury is attenuated by inhibition of oxidative stress and mitochondrial respiratory dysfunction^10^, as well as stimulation to restore mitochondrial mass and membrane potential^10,11^. Here, we investigated the effect of nobiletin on autophagy and mitochondrial regulation during hepatic IR.

Sirtuin-1 (SIRT-1) is an important metabolic sensor that responds to cellular stress, starvation, and caloric restriction^12^. Activation of SIRT-1 promotes the transcription of genes that regulate mitochondrial biogenesis to maintain energy and metabolic homeostasis^13,14^. Indeed, SIRT-1 is the principal target of many phytoflavonoids, including resveratrol, which has beneficial metabolic and antiaging effects^15^. SIRT-1 activation by such phytochemicals regulates peroxisome proliferator-activated receptor gamma coactivator-1 alpha (PGC-1α)-mediated mitochondrial biogenesis and mitophagy. In addition, SIRT-1 deacetylates members of the forkhead box O (FoxO) family and affects downstream pathways controlling autophagy^16,17^. Pharmacological stimulation of SIRT-1 attenuates hepatic IR injury through mitochondrial recovery and enhanced autophagy^11^. Thus, we aimed to determine whether nobiletin protects against hepatic IR through SIRT-1.

**Fig. 1:**
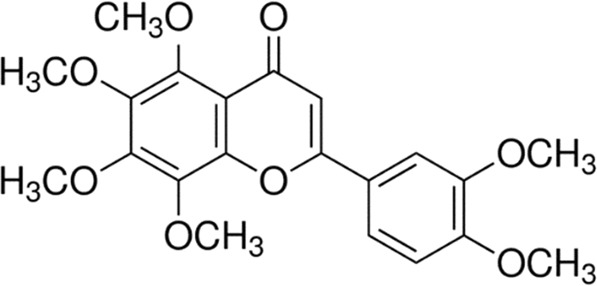
Chemical structure of nobiletin

Nobiletin (Fig. 1) is a polymethoxyflavone primarily present in citrus fruits and has a broad range of beneficial properties, including antioxidant, anti-inflammatory^18,19^, anti-cancer^20^, and antidiabetic^21^ activities. Nobiletin reduces cerebral ischemic injury by upregulating nuclear factor (erythroid-derived 2)-like-2 (Nrf2) and HO-1, attenuates IR injury following liver transplantation through the suppression of Kupffer cell activation, and protects against acute myocardial infarction by restoring impaired autophagic flux^19,22,23^. However, the detailed molecular mechanisms by which nobiletin regulates autophagy and mitochondrial function in hepatic IR injury remain undefined. Thus, we investigated the effect of nobiletin on hepatic IR injury and its underlying molecular mechanisms.

## Materials and methods

### Experimental animals

Male C57BL/6 mice (9-week-old) were purchased from Koatech (Pyeongtaek, Korea) and maintained in the animal facility at Gyeongsang National University (GNU). All animal experiments were approved by the Institutional Board of Animal Research at GNU and performed in accordance with the National Institutes of Health guidelines for laboratory animal care. Mice were housed under alternating 12 -h light/dark cycles with a relative humidity of 60 ± 10% and a controlled temperature of 22 ± 2°C. Animals were provided with standard chow ad libitum and tap water.

### Animal model of hepatic IR

Mice were randomly divided into five groups of six mice each: sham, hepatic IR (HIR) treated with the vehicle or nobiletin, and HIR pretreated with a SIRT-1 inhibitor and treated with the vehicle or nobiletin. The SIRT-1 inhibitor, EX-527, was administered intraperitoneally (i.p.) once daily for 3 days prior to ischemia. Anesthetized mice were placed supine on a heating pad under a heat lamp to maintain body temperature throughout the surgical procedures. After a midline abdominal incision, the liver hilum was exposed, and the hepatic artery and portal vein were identified. A microaneurysm clip was placed across the hilum of the left and median lobes to produce a 70% hepatic warm ischemia. The right lobes remained perfused to prevent intestinal blood congestion. After 60 min of ischemia, the clip was removed, and the abdomen was closed with sutures. Nobiletin (5 mg/kg) or the vehicle (0.025% carboxymethyl cellulose) was i.p. injected at the start of reperfusion. The nobiletin dose was determined based on a pilot study (Supplementary Fig. 1). Blood and ischemic hepatic tissues were collected at 5 h of reperfusion. The median and left lobes were immediately removed and either fixed in 10% formalin or snap-frozen in liquid nitrogen and stored at −80 °C.

### Biochemical assays

Plasma alanine aminotransferase (ALT) and aspartate aminotransferase (AST) were measured using a commercial assay kit (IVD Lab Co., Ltd., Uiwang, Korea) and a spectrophotometer (Shimadzu UV-1800 spectrophotometer, Tokyo, Japan). Hepatic glutathione (GSH) levels were assayed from liver tissue lysates using a GSH/glutathione disulfide (GSSG) Ratio Detection Kit II (Fluorometric-Green, Abcam 205811; Cambridge, UK) according to the manufacturer’s instructions, and the wavelength was determined (Ex/Em = 490/520 nm) using a Tecan Infinite M200 PRO Microplate Reader (Tecan Austria GmbH, Grödig, Austria).

### Histological evaluation of liver injury

The fixed liver tissues were embedded in paraffin, and the blocks were cut into 5 -μm sections. The sections were then stained with hematoxylin and eosin (H&E) according to standard procedures and examined using a Nikon Eclipse Ti-U microscope (Tokyo, Japan). The histological injury score for each sample was expressed as the sum of the individual scores for three different parameters based on the following Suzuki criteria: congestion (None = 0, Minimal = 1, Mild = 2, Moderate = 3, Severe = 4), vacuolization (None = 0, Minimal = 1, Mild = 2, Moderate = 3, Severe = 4), and necrosis (None = 0, Single Cell Necrosis = 1, < 30% = 2, 30–60% = 3, > 60% = 4); scores for each parameter ranged from 0 to 4, with a maximum score of 12^24^.

### Terminal deoxynucleotidyl transferase dUTP nick-end labeling assay

Terminal deoxynucleotidyl transferase dUTP nick-end labeling (TUNEL) staining was performed to evaluate the degree of apoptosis using an In Situ Cell Death Fluorescein Detection Kit (Roche Molecular Biochemicals, Mannheim, Germany) according to the manufacturer’s instructions. The images were captured using a Nikon Eclipse Ti-U microscope (Tokyo, Japan).

### Western blot analysis

Liver tissues were homogenized in ice-cold radioimmunoprecipitation assay (RIPA) buffer with protease inhibitors (Thermo Fisher Scientific, Waltham, MA, USA), sonicated, and incubated for 30 min on ice. After centrifugation, the supernatant was transferred to a clean tube, and the protein concentration was determined using a Pierce^TM^ BCA Protein Assay Kit (Thermo Fisher Scientific). The protein lysates were separated using SDS-PAGE and transferred to PVDF membranes. After blocking with 5% skim milk, the membranes were incubated with primary antibodies against SIRT-1, FOXO3a, phosphorylated AMP-activated protein kinase (p-AMPK), AMPK, p-AKT, AKT, uncleaved caspase-3, cleaved caspase-3, uncleaved poly (ADP-ribose) polymerase 1 (PARP-1), cleaved PARP-1, autophagy-related gene5 (ATG5), ATG7, ATG12, light-chain 3B (LC3B), and P62 (Cell Signaling Technology, Danvers, MA, USA); dynamin-related protein 1 (DRP-1; Santa Cruz Biotechnology, Dallas, TX, USA); PGC-1α, p-FOXO3a, mitofusin 2 (MFN-2), 4-hydroxynonenal (4-HNE), matrix metalloproteinase 2 (MMP-2), MMP-9, and p-84 (Abcam); optic atrophy 1 (OPA-1; BD Biosciences, Franklin Lakes, NJ, USA); and β-actin (Sigma, St. Louis, MO, USA) in blocking solution at 4 ^○^C overnight. The membranes were then incubated with the appropriate horseradish peroxidase (HRP)-conjugated secondary antibodies (Bio-Rad, Hercules, CA, USA) at room temperature for 1 h and then visualized with the ECL substrate (Bio-Rad). The ChemiDoc XRS + System (Bio-Rad) was used to evaluate the density of the protein bands. The relative protein expression was quantified using Image Lab^TM^ software (Bio-Rad).

### Nuclear–cytoplasmic fractionation

Nuclear–cytoplasmic fractionation was conducted using NE-PER^TM^ nuclear and cytoplasmic extraction reagents (Life Technologies, 78855, Carlsbad, CA, USA) according to the manufacturer’s instructions. The protein concentration was determined using a Pierce^TM^ BCA Protein Assay Kit (Thermo Fisher Scientific), and equal amounts of proteins were separated by SDS-PAGE. To assess fractionation efficiency, cytoplasmic and nuclear fractions were confirmed by immunoblotting with anti-β-tubulin (Sigma T8328) and anti-P84 (Abcam 46545), respectively.

### Quantitative real-time PCR (qRT-PCR) analysis

The total RNA was extracted with TRIzol (Invitrogen, Carlsbad, CA, USA) and converted into cDNA using the RevertAid Reverse Transcription System (Thermo Fisher Scientific), according to the manufacturer’s protocol. qPCR was performed with a CFX Connect Real-Time PCR System using iQ SYBR Green Supermix (Bio-Rad). Relative mRNA levels were normalized to those of GAPDH. The primer sequences are listed in Table 1.

**Table 1:** The primer sequences used for qRT-PCR analysis in this study

Gene	Primers (5′-3′)
ATG5	Forward: ACCAAATCGTTACATATTCC
	Reverse: CAAGGGTTCTTCTAAACTTG
ATG7	Forward: CGCTTGACGTTGGAGTTCAGTG
	Reverse: GTGTTGTGCAGGGTTCCCATG
ATG12	Forward: AGCTCTTCAGTCCTGTCATTTC
	Reverse: ACTCCTGGTTCACTCTTCCT
Beclin-1	Forward: GTCTAAGGCGTCCAGCAGCAC
	Reverse: TGGGCTGTGGTAAGTAATGGAGC
DRP-1	Forward: ACCAAAGTACCTGTAGGCGATC
	Reverse: CATGGCATCAGTACCCGCAT
FOXO3a	Forward: CTGTCCTATGCCGACCTGATCAC
	Reverse: CATTCTGAACGCGCATGAAGCG
IL-1β	Forward: TTTGTACAAGGAGAACCAAG
	Reverse: TTTCATTACACAGGACAGGT
IL-6	Forward: CCAATTCATCTTGAAATCAC
	Reverse: GGAATGTCCACAAACTGATA
MFN-2	Forward: ACCGTCAAGAAGGATAAGCGACAC
	Reverse: GTGTTCCTGTGGGTGTCTTCAAGG
MMP-2	Forward: GTTCAACGGTCGGGAATACA
	Reverse: GCCATACTTGCCATCCTTCT
MMP-9	Forward: CTGGAACTCACACGACATCTT
	Reverse: TCCACCTTGTTCACCTCATTT
NRF1	Forward: GAGCACGGAGTGACCCAAAC
	Reverse: TGTACGTGGCTACATGGACCT
OPA-1	Forward: CCAAGAACGAGTTGGAGAAGATGC
	Reverse: CACGTCATTGCATTCCAGCTCAGA
PGC-1α	Forward: AGCCGTGACCACTGACAACGAG
	Reverse: GCTGCATGGTTCTGAGTGCTAAG
SIRT-1	Forward: ACCAAATCGTTACATATTCC
	Reverse: CAAGGGTTCTTCTAAACTTG
TFAM	Forward: CCAAGTCAGCTGATGGGTATGG
	Reverse: CCTGAGCCGAATCATCCTTTGC
TNFα	Forward: GAGCAATGACTCCAAAGTAG
	Reverse: CCAATTCATCTTGAAATCAC

### Statistical analysis

Statistical differences among the groups were determined using one-way analysis of variance followed by Bonferroni post hoc analysis. The values were expressed as the mean ± SEM. A *p-*value < 0.05 was considered statistically significant.

## Results

### Nobiletin attenuated hepatocellular damage induced by hepatic IR injury

**Fig. 2:**
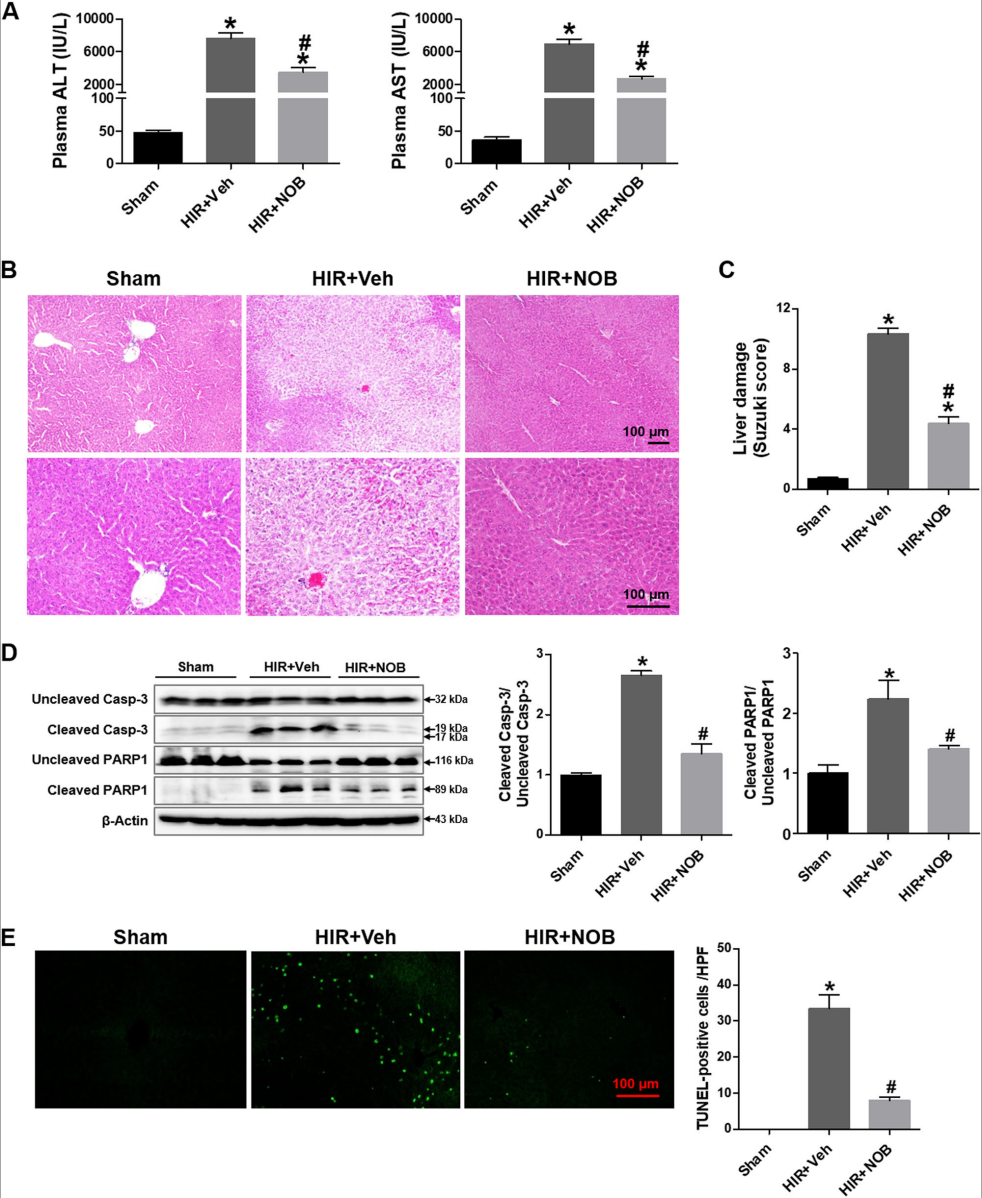
Effect of nobiletin on hepatocellular damage during hepatic IR injury. Mouse livers were subjected to ischemia for 60 min followed by 5 h of reperfusion. The mice were treated with the vehicle or nobiletin (5 mg/kg) at the start of reperfusion. The liver tissues and blood were harvested from sham-operated (sham group) or hepatic IR-subjected (HIR group) mice. **a** Plasma ALT and AST levels were measured to assess the degree of liver injury. **b** Representative images of hematoxylin and eosin (H&E) staining from liver sections to evaluate hepatic necrosis and pathological changes. **c** The extent of liver damage was graded by Suzuki score as described in the Materials and methods section. **d** The degree of hepatocellular apoptosis was determined by caspase-3 and PARP-1 cleavage in liver tissue lysates using western blot analysis, and quantitative analysis is shown. **e** Apoptosis-positive cells were analyzed by TUNEL, and the numbers of apoptotic cells per ×200 field image were counted. The data are presented as the mean ± SEM (*n* = 6). *p < 0.05 vs. Sham group, ^#^*p* < 0.05 vs. HIR + Veh group. Scale bar, 100 μm

Liver damage was assessed by measuring plasma ALT and AST, which were significantly increased in the hepatic IR group compared with the sham group (Fig. 2a). In contrast, nobiletin treatment at the start of reperfusion significantly decreased ALT and AST levels by 53.9% and 64.3%, respectively, compared with the hepatic IR group (Fig. 2a). These results were consistent with histopathological changes in liver tissues. Liver tissues from the hepatic IR group demonstrated increased necrotic area, sinusoidal congestion, and cytoplasmic vacuolization of hepatocytes compared with the liver tissues of the sham group. These features were significantly attenuated by nobiletin treatment (Fig. 2b). The extent of liver damage was quantitatively presented based on the Suzuki classification (Fig. 2c).

To investigate the effect of nobiletin on hepatic apoptosis induced by IR injury, we performed a western blot analysis to assess cleavage of caspase-3 and PARP-1. Cleavage of caspase-3 and PARP-1 significantly increased in the hepatic IR group compared with the sham group, which was attenuated by nobiletin treatment (Fig. 2d). TUNEL staining showed a significant increase in the number of TUNEL-positive cells after IR injury, which was decreased upon nobiletin treatment (Fig. 2e).

### Nobiletin decreased oxidative stress and induction of inflammatory cytokines and MMPs induced by hepatic IR injury

**Fig. 3:**
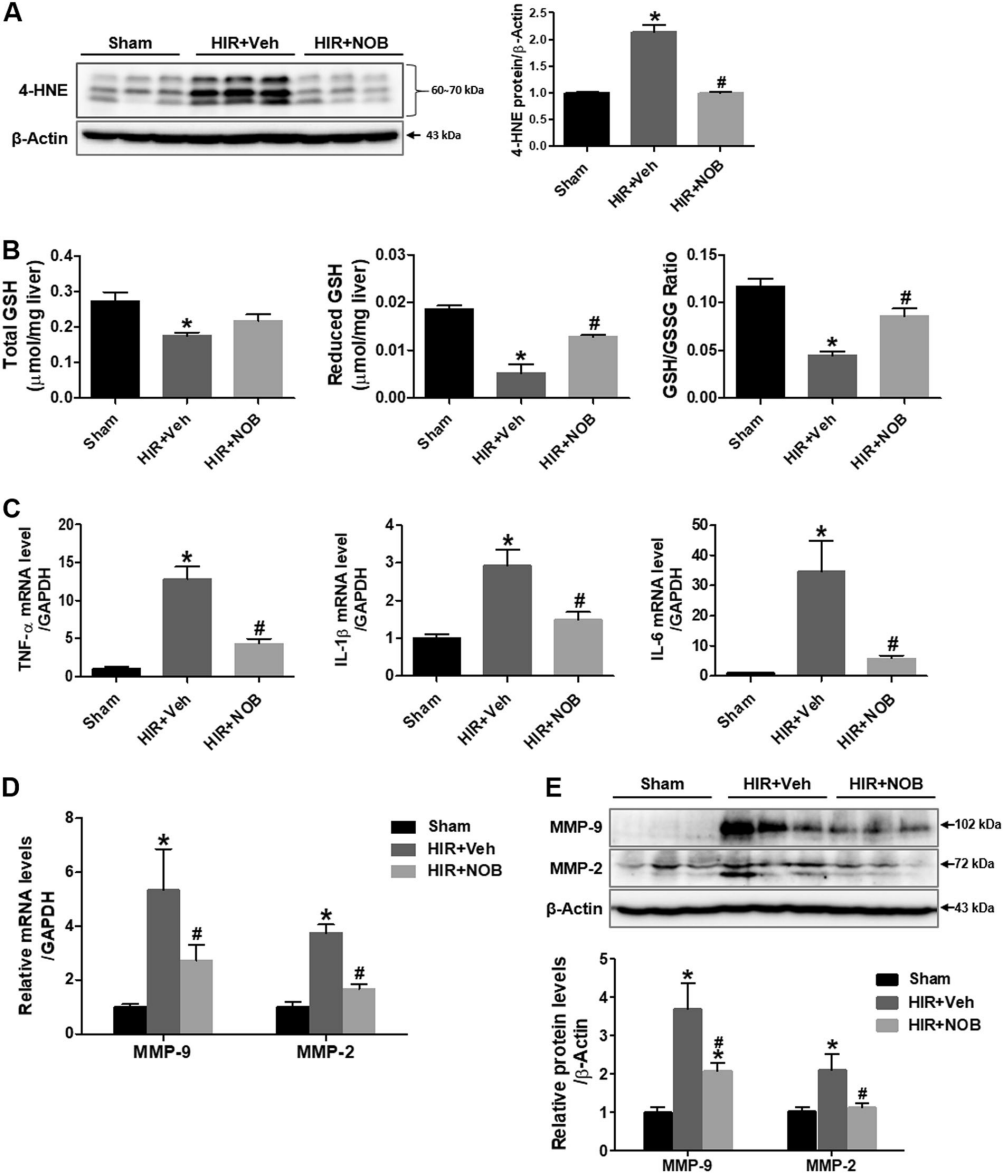
Effect of nobiletin on oxidative stress and proinflammatory cytokine and matrix metalloproteinase (MMP) expression during hepatic IR injury. **a** 4-Hydroxynonenal (4-HNE) protein levels were assessed by western blotting, and quantitative analysis is shown. **b** The total GSH, reduced GSH, and GSH/GSSH ratios were measured in liver tissue lysates by using a colorimetric assay. **c** The mRNA expression of tumor necrosis factor-α (TNF-α), interleukin-1β (IL-1β), and interleukin-6 (IL-6) was determined in liver tissues by real-time PCR analysis. **d** The mRNA expression level of matrix metalloproteinase (MMP)-2 and MMP-9 was determined in liver tissues by real-time PCR analysis. Relative mRNA expression was normalized to that of GAPDH. **e** MMP-2 and MMP-9 protein levels were assessed by western blot analysis in liver tissue lysates, and the relative expression was determined by using β-actin as a loading control. The data are presented as the mean ± SEM (*n* = 6). *p < 0.05 vs. Sham group, ^#^*p* < 0.05 vs. HIR + Veh group. Scale bar, 100 μm

Oxidative stress was then assessed by 4-HNE, a marker of lipid peroxidation. The levels of 4-HNE increased significantly in the hepatic IR group compared with the sham group, which decreased upon nobiletin treatment (Fig. 3a). Hepatic levels of the total and reduced GSH and GSH/GSSG ratios were assessed as indicators of endogenous antioxidant system activity. We found that the total GSH decreased after IR, but there was no significant increase upon nobiletin treatment. However, the reduced GSH and GSH/GSSG ratio markedly increased upon nobiletin treatment compared with the hepatic IR group (Fig. 3b).

We next investigated whether nobiletin reduces inflammatory cytokine and MMP expression during hepatic IR using real-time PCR analysis. Hepatic IR-induced mRNA expression of the proinflammatory cytokines tumor necrosis factor-α (TNF-α), interleukin-1β (IL-1β), and interleukin-6 (IL-6) was significantly reduced upon nobiletin treatment (Fig. 3c). Similarly, upregulation of MMP-9 and MMP-2 genes was reduced upon nobiletin treatment (Fig. 3d). Furthermore, western blot analysis demonstrated a significant increase in MMP-2 and MMP-9 protein levels in the hepatic IR group, which was reduced by nobiletin treatment (Fig. 3e).

### Nobiletin attenuated hepatic IR injury through induction of autophagy

**Fig. 4:**
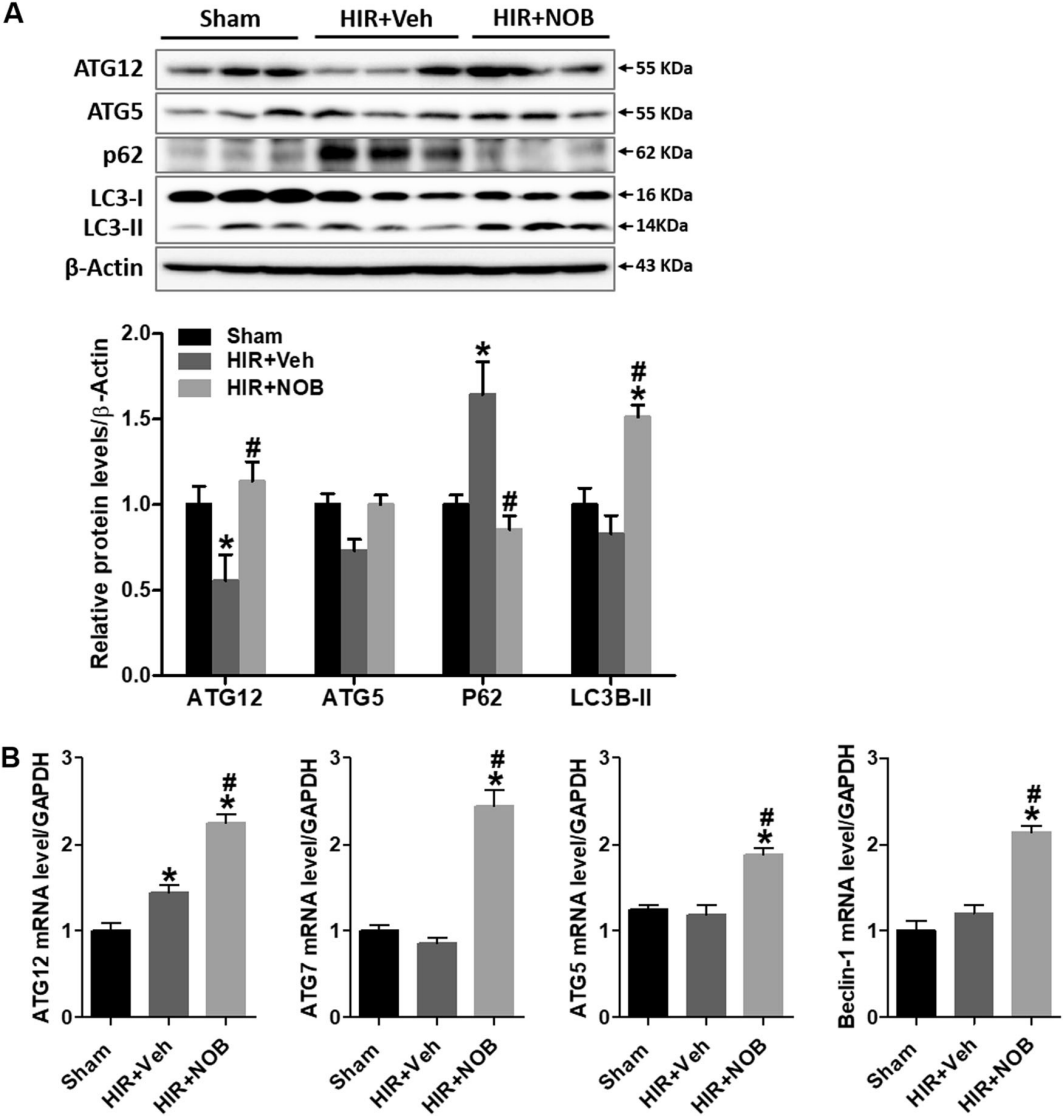
Effect of nobiletin on autophagy response during hepatic IR injury. **a** The autophagy-related proteins ATG5, ATG12, LC3B, and p62 were examined by western blot analysis in liver tissue lysates, and relative protein expression was determined by using β-actin as a loading control. **b** The mRNA expression of ATG5, ATG7, ATG12, and Beclin-1 was determined in liver tissues by real-time PCR analysis. Relative mRNA expression was normalized to that of GAPDH. The data are presented as the mean ± SEM (*n* = 6). *p < 0.05 vs. Sham group, ^#^*p* < 0.05 vs. HIR + Veh group

We next investigated whether nobiletin induces autophagy in the liver following hepatic IR injury. The levels of autophagy-related proteins (ATG5, ATG12, and LC3B-II) decreased, and p62, a marker of autophagy deficit, significantly increased in the hepatic IR group compared with the sham group. In contrast, nobiletin treatment markedly increased the expression of autophagy-related proteins and decreased p62 protein accumulation in the liver following IR injury compared with the hepatic IR group (Fig. 4a). Similarly, nobiletin treatment significantly induced autophagy-regulatory genes (ATG5, ATG12, ATG7, and Beclin-1) during hepatic IR injury (Fig. 4b).

### Nobiletin protected against mitochondrial dysfunction by restoring mitochondrial biogenesis and fusion–fission dynamics in hepatic IR injury

**Fig. 5:**
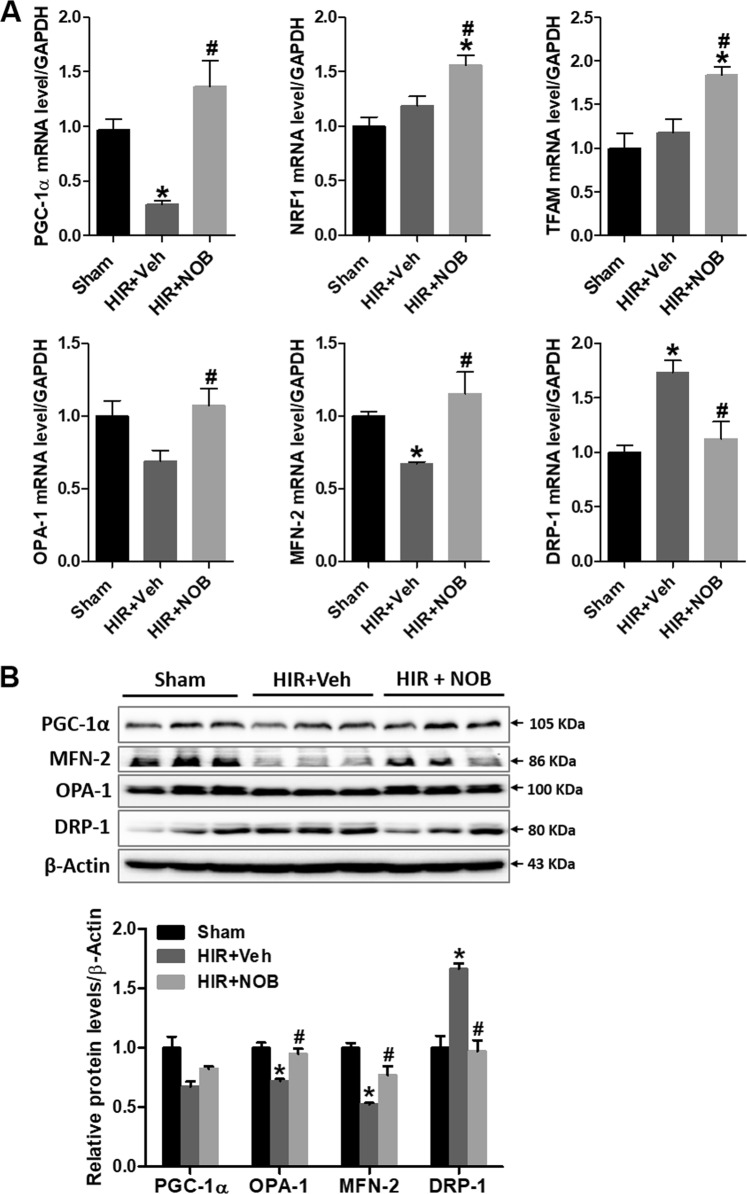
Effect of nobiletin on mitochondrial biogenesis and fusion–fission dynamics during hepatic IR injury. **a** The mRNA expression of PGC-1α, NRF1, TFAM, MFN-2, OPA-1, and DRP-1 was determined in liver tissues by real-time PCR analysis. Relative mRNA expression was normalized to that of GAPDH. **b** The protein expression level of PGC-1α, MFN-2, OPA-1, and DRP-1 was assessed in liver tissue lysates by western blotting, and the relative expression was determined by using β-actin as a loading control. Data are presented as the mean ± SEM (*n* = 6). *p < 0.05 vs. Sham group, ^#^*p* < 0.05 vs. HIR + Veh group

Mitochondrial biogenesis is tightly regulated by PGC-1α and the downstream nuclear respiratory factor 1 (NRF1) and mitochondrial transcription factor A (TFAM) pathways, which control mitochondrial turnover, content, and number to maintain diverse metabolic demands^25,26^. In this study, we found that mRNA expression of PGC-1α, NRF1, and TFAM was significantly increased upon nobiletin treatment in the hepatic IR group compared with the sham group (Fig. 5a). Moreover, mRNA expression of MFN-2 increased and DRP-1 expression decreased; however, OPA-1 expression was not significantly changed (Fig. 5a). We also found that protein expression of PGC-1α, MFN-2, and OPA-1 was decreased, whereas DRP-1 expression was increased in the hepatic IR group, which was alleviated upon nobiletin treatment (Fig. 5b). These results indicate that nobiletin protects against IR-induced mitochondrial dysfunction by regulating PGC-1α-mediated mitochondrial biogenesis and balancing fusion–fission dynamics.

### Nobiletin increased SIRT-1 and FOXO3a expression in hepatic IR injury

**Fig. 6:**
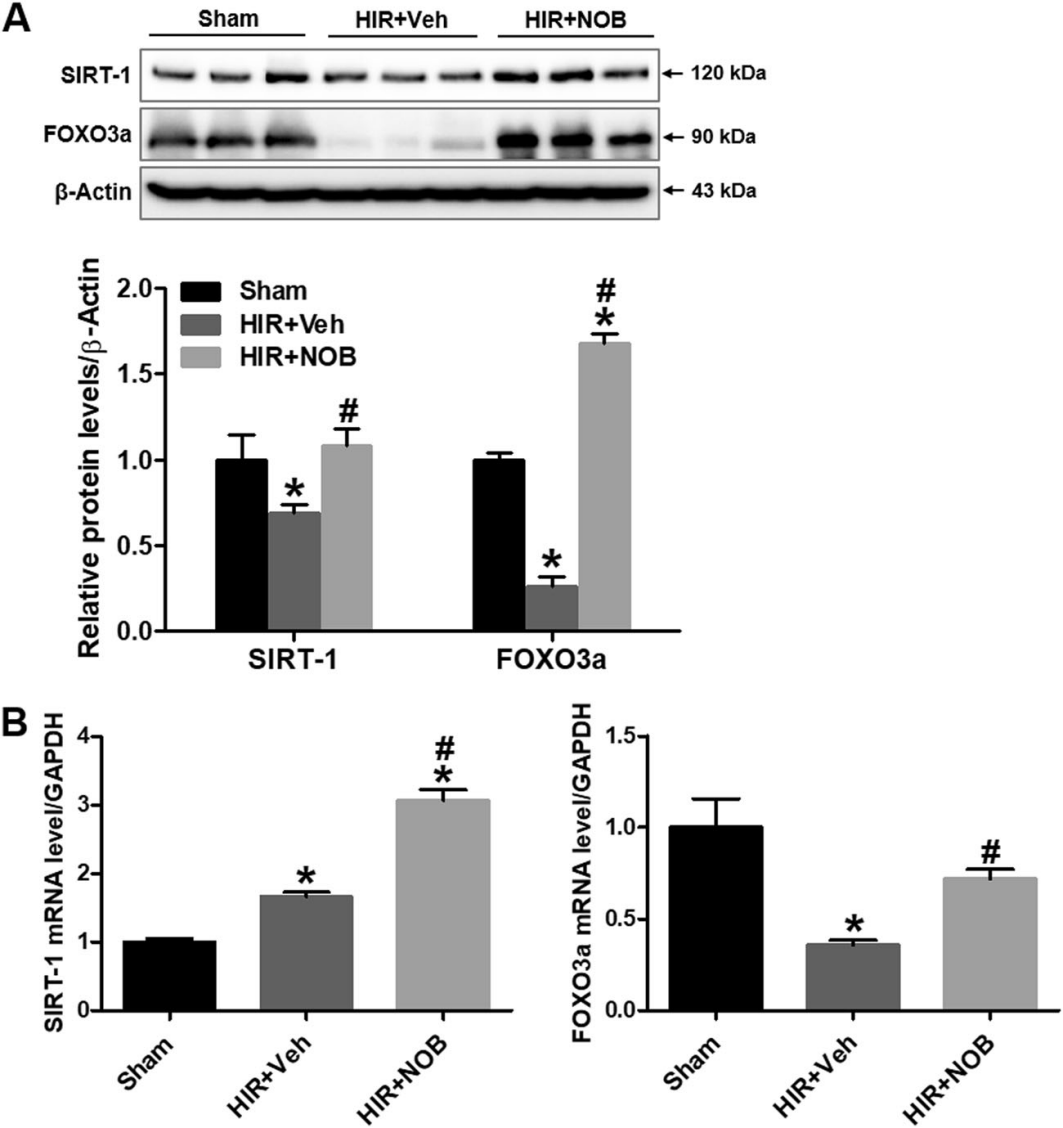
Effect of nobiletin on SIRT-1 and FOXO3a expression during hepatic IR injury. **a** SIRT-1 and FOXO3a protein expression was examined by western blot analysis in the liver tissue lysates, and relative expression was determined by using β-actin as a loading control. **b** The mRNA expression of SIRT-1 and FOXO3a was determined in liver tissue lysates by real-time PCR analysis. Relative mRNA expression was normalized to that of GAPDH. The data are presented as the mean ± SEM (*n* = 6). *p < 0.05 vs. Sham group, ^#^*p* < 0.05 vs. HIR + Veh group

Considering that SIRT-1/FOXO3a signaling plays a key role in autophagy induction^27^, we examined whether nobiletin affects SIRT-1 and FOXO3a expression in hepatic IR mice. We found that SIRT-1 and FOXO3a protein levels decreased in the hepatic IR group compared with the sham group, and nobiletin treatment significantly increased SIRT-1 and FOXO3a protein levels (Fig. 6a). Furthermore, qRT-PCR analysis revealed a significant increase in SIRT-1 and FOXO3a mRNA after nobiletin treatment in hepatic IR mice (Fig. 6b).

### Nobiletin decreased AKT phosphorylation to promote FOXO3a nuclear expression in hepatic IR injury

**Fig. 7:**
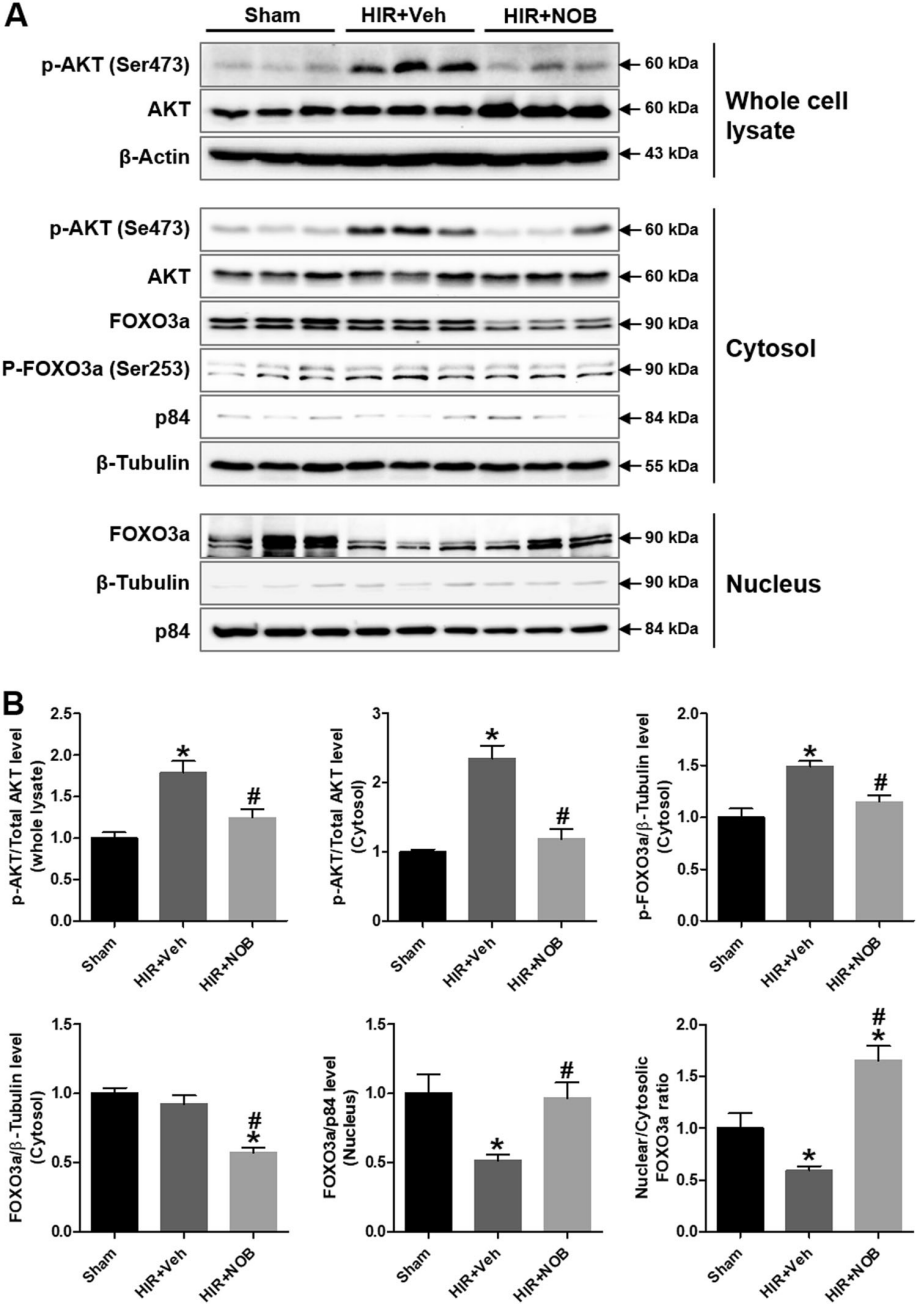
Effect of nobiletin on AKT phosphorylation and FOXO3a expression during hepatic IR injury. Whole-liver tissue lysates and the nuclear and cytosolic fractions were prepared to evaluate AKT phosphorylation and FOXO3a localization. **a** The protein expression of AKT, p-AKT (Ser473), FOXO3a, and p-FOXO3a (Ser253) was examined by western blot analysis. **b** The relative expression was determined by using total AKT, β-tubulin (cytosolic marker), and p84 (nuclear marker) as loading controls. The data are presented as the mean ± SEM (*n* = 6). *p < 0.05 vs. Sham group, ^#^*p* < 0.05 vs. HIR + Veh group

FOXO3a plays a critical role in regulating autophagy, apoptosis, and oxidative stress in various disease conditions. In response to stress or pathological conditions, FOXO3a is phosphorylated by AKT and is retained in the cytosol, thereby inhibiting its transcriptional activity^28,29^. Activated FOXO3a increases ATG gene transcription in mouse alcoholic livers^30^ and atrophying muscle cells^31^. We therefore investigated the mechanism of nobiletin-mediated protection through the AKT and FOXO3a pathways (Fig. 7). The hepatic IR group exhibited a significant increase in AKT phosphorylation, which was reduced upon nobiletin treatment in whole-cell lysates. We then determined whether AKT phosphorylation levels were correlated with changes in the subcellular localization of FOXO3a. Analysis of cytosolic and nuclear fractions revealed that the nuclear versus cytosolic FOXO3a was significantly increased by its nuclear translocation from the cytosol following nobiletin treatment, which was coincident with a reduction in AKT phosphorylation and FOXO3a expression. These results suggest that nobiletin increases FOXO3a nuclear retention and subsequent transcriptional activity by inhibiting IR-induced AKT phosphorylation.

### SIRT-1 inhibition abolished the protective effect of nobiletin against hepatic IR injury

**Fig. 8:**
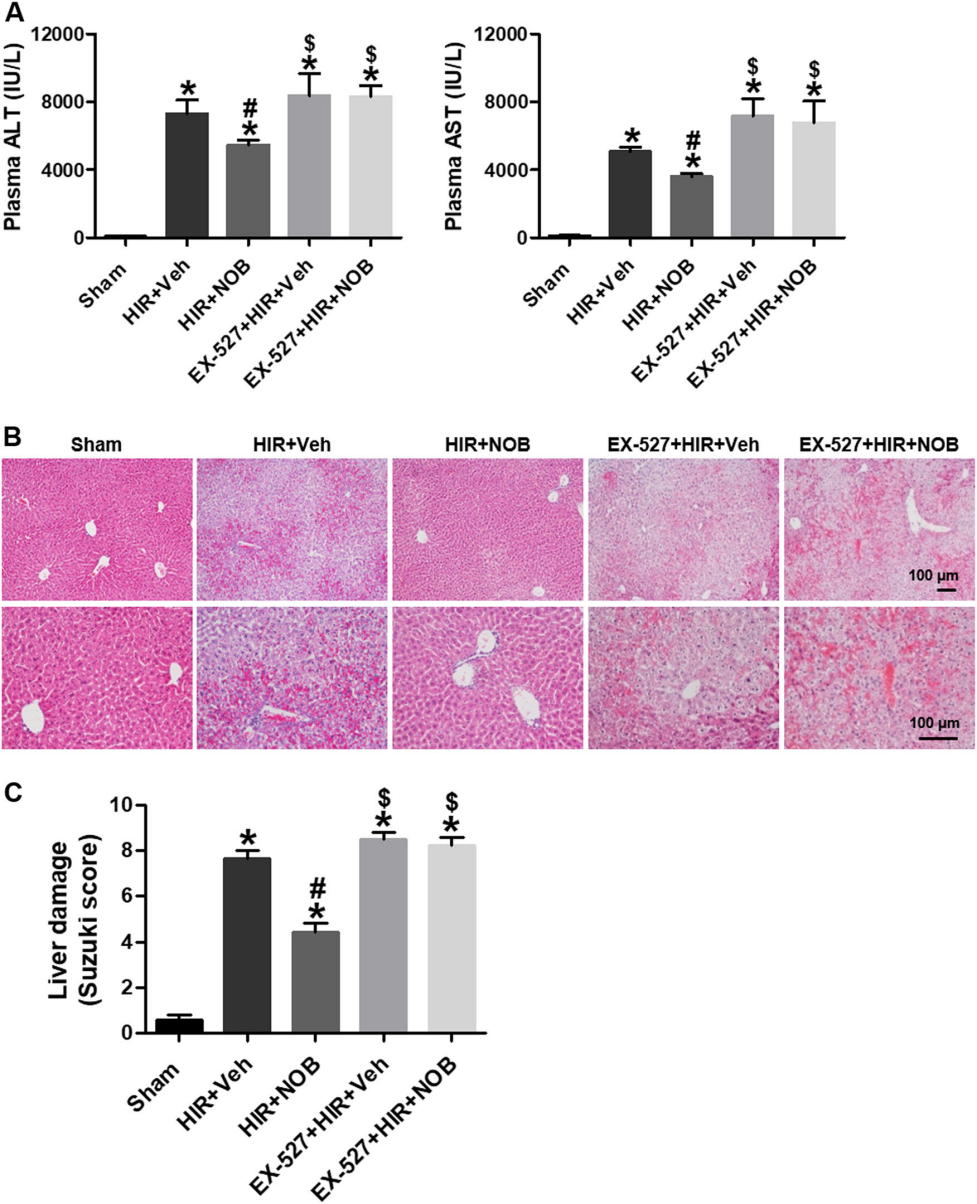
Effect of SIRT-1 inhibition on nobiletin-mediated protection against hepatocellular damage during hepatic IR injury. Mouse livers were subjected to ischemia for 60 min followed by 5 h of reperfusion. The mice were treated with vehicle or SIRT-1 inhibitor EX527 (5 mg/kg) for 3 days before ischemia and then treated with nobiletin (5 mg/kg) at the start of reperfusion. The liver tissues and blood were harvested after reperfusion. Plasma ALT and AST levels (**a**), representative images of H&E staining (**b**), and liver damage based on the Suzuki scoring system (**c**) are shown. The data are presented as the mean ± SEM (*n* = 6). *p < 0.05 vs. Sham group, ^#^*p* < 0.05 vs. HIR + Veh group, ^$^*p* < 0.05 vs. HIR + NOB group. Scale bar, 100 μm

A specific SIRT-1 inhibitor (EX-527) was used to investigate whether SIRT-1 activity is required for nobiletin-mediated protection against hepatic IR injury. We found that plasma ALT and AST were reduced in the nobiletin-treated hepatic IR group, and this effect was blocked by EX-527 treatment (Fig. 8a). H&E staining further demonstrated that EX-527 treatment abolished the protective effect of nobiletin on reducing hepatic necrosis and corresponding Suzuki scores (Fig. 8b, c). Interestingly, EX-527-induced SIRT-1 inhibition often exacerbated IR-induced hepatic damage. These results indicate that SIRT-1 activity is required for a nobiletin-mediated decrease in hepatocellular damage following hepatic IR injury.

**Fig. 9:**
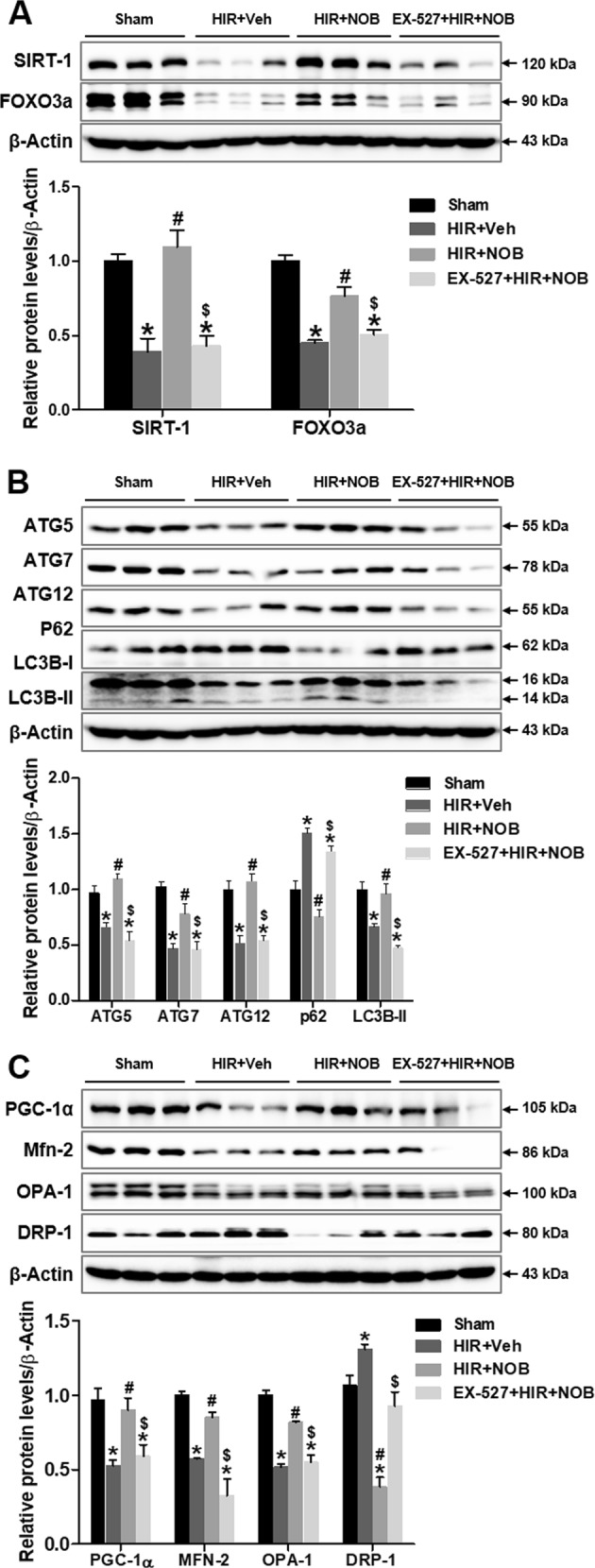
Effect of SIRT-1 inhibition on nobiletin-mediated autophagy induction and mitochondrial biogenesis and dynamics during hepatic IR injury. Whole-liver tissue lysates were prepared to examine protein expression levels by western blot analysis; the blots of SIRT-1, FOXO3a (**a**), autophagy-related proteins ATG5, ATG7, ATG12, LC3B, p62 (**b**), and mitochondria-regulating proteins PGC-1α, MFN-2, OPA-1, DRP-1 (**c**) are shown. The relative expression was determined by using β-actin as a loading control. The data are presented as the mean ± SEM (*n* = 6). *p < 0.05 vs. Sham group, ^#^*p* < 0.05 vs. HIR + Veh group, ^$^*p* < 0.05 vs. HIR + NOB group

SIRT-1 stimulation attenuates hepatic IR injury by activating PGC-1α to promote mitochondrial biogenesis/recovery and deacetylating FOXO3a to enhance autophagy^11,27,30^. We demonstrated that nobiletin upregulated SIRT-1, with a simultaneous increase in FOXO3a expression. However, the effect was significantly blocked by SIRT-1 inhibition (Fig. 9a). FOXO3a-mediated induction of autophagy-related proteins (ATGs and LC3B-II) was attenuated by EX-527 treatment, with significant accumulation of p62 (Fig. 9b). PGC-1α and mitochondrial fusion versus fission was upregulated by nobiletin, but the effect was abolished by EX-527. These results indicate that SIRT-1 activity is specifically required for FOXO3a-mediated induction of autophagy, PGC-1α-mediated mitochondrial biogenesis, and balancing mitochondrial fusion–fission dynamics, resulting in alleviation of hepatic IR injury (Fig. 9c). A summarized molecular mechanism of the nobiletin effect is depicted in Supplementary Fig. 2, illustrating that SIRT-1 is an essential upstream regulator of PGC-1α and FOXO3a, enhancing mitochondrial and autophagy function and protecting the liver against hepatic IR injury.

## Discussion

We demonstrated that nobiletin attenuates hepatic IR injury in the following ways: (1) nobiletin enhances autophagy and mitochondrial biogenesis and dynamics, (2) nobiletin induces autophagy through a SIRT-1/FOXO3a signaling pathway, (3) nobiletin-mediated control of mitochondrial biogenesis depends on a PGC-1α/NRF1/TFAM signaling pathway, (4) nobiletin improves mitochondrial dynamics by regulating the expression of fusion and fission proteins, and (5) nobiletin reduces oxidative damage, the expression of MMPs and inflammation.

Hepatic IR injury is the primary cause of acute or chronic liver damage after liver transplantation or dissection, and is characterized by oxidative stress, inflammation, disruption of epithelial integrity, and impaired autophagy and mitochondrial function. These changes lead to hepatocyte necrosis and apoptosis, which contribute to organ failure^32^. We propose that enhancing autophagy and mitochondrial function is a promising therapeutic strategy to attenuate IR-induced damage.

Nobiletin, chemically 5,6,7,8,3′,4′-hexamethoxyflavone, is a flavonoid abundant in citrus peel that has demonstrated various pharmacological activities against cardiovascular and metabolic disorders, inflammation, and cancers^33^. The biological activities of nobiletin depend on its chemical structure; nobiletin is absorbed easily due to its lipophilic nature and high permeability. An X-ray crystal structure study revealed that the chromene and arene rings and the bound methoxy groups create a chiral molecule that can adopt a variety of conformations^34^. This molecular flexibility may support a wide range of physiological activities by binding the different target receptors of specific signaling pathways.

Since nobiletin is known as an MMP inhibitor, we first determined the effect of nobiletin on MMP-2 and -9 expression during hepatic IR injury. MMPs, proteolytic enzymes that degrade the extracellular matrix proteins, have been linked to cancer and chronic inflammation^35^. MMP-9 is induced by leukocytes following hepatic IR and is important for leukocyte migration into inflamed livers and subsequent cytokine release^36^. MMP-2 plays an important role in hepatic vascular homeostasis, is expressed in human fibrotic livers, and can either promote or inhibit inflammation in a condition-dependent manner^37^. Interestingly, an MMP-2 deletion enhances MMP-9 activity and exacerbates hepatic IR injury^38^, while MMP-9 deletion protects against hepatic IR injury in mice^39^. Treatment with bortezomib, a proteasome inhibitor, protects against steatotic liver IR injury by inhibiting MMP-2 and MMP-9 activation and reducing proinflammatory cytokines^40^. Similarly, our results showed that nobiletin inhibited expression of both MMP-2 and MMP-9 and downregulated proinflammatory cytokines following hepatic IR. The molecular mechanism of nobiletin-mediated regulation of MMPs requires further study.

Mitochondrial dysfunction is a central feature of IR injury, as mitochondria are important for energy homeostasis and directly mediate oxidative stress and cell death^41^. Mitochondria play a key role in initiating apoptosis through a permeability transition followed by excessive ATP depletion and ROS production, and mitochondrial breakdown leads to necrosis and apoptosis^42^. In this study, nobiletin attenuated oxidative stress and hepatic cell death induced by necrosis and apoptosis during hepatic IR injury.

Mitochondrial biogenesis is a critical control mechanism for mitochondrial turnover, content, and number, all of which are needed to meet metabolic energy demands and to prevent cell death in various diseases^25^. PGC-1α is a major regulator of mitochondrial biogenesis that activates different transcription factors, including NRF1 and TFAM. NRF1 is a transcription factor that stimulates mitochondrial enzyme expression and directly interacts with TFAM to promote transcription and replication of mitochondrial DNA (mtDNA)^43^. Therefore, we examined PGC-1α, NRF1, and TFAM expression, which positively regulates mitochondrial biogenesis. Recent studies found that cilostazol or genipin treatment stimulates mitochondrial biogenesis via induction of the PGC-1α/NRF1/TFAM pathway, leading to improved mitochondrial functioning during hepatic IR injury^26,44^. Similarly, in this study, hepatic IR decreased PGC-1α, NRF1, and TFAM expression, but these factors were significantly upregulated upon nobiletin treatment. Nobiletin has the potential to enhance mitochondrial biogenesis and protect against IR-induced mitochondrial dysfunction.

SIRT-1 plays an important role in many physiological processes of cellular energetics, metabolism, and aging, in particular by interacting with PGC-1α^13,45^, SIRT-1 interacts with and deacetylates PGC-1α to increase its activity and control mitochondrial function. SIRT-1 activation reduces IR-induced oxidative damage by enhancing mitochondrial biogenesis and restoring impaired mitophagy and mitochondrial dynamics^26^. Similarly, our results showed that nobiletin increased SIRT-1 expression and upregulated PGC-1α to promote mitochondrial biogenesis and dynamics, thus attenuating hepatic IR injury.

Mitochondrial dynamics play a critical role in determining mitochondrial morphology and function. Fusion–fission dynamics are regulated by the expression and activity of the fusion (MFN-1/2 and OPA-1) and fission (DRP-1) proteins^46^. DRP-1 inhibition protects against mitochondrial fragmentation and apoptosis in mice with senecionine-induced liver injury, while reduced endogenous MFN-2 expression impairs mitochondrial fusion, contributing to the pathogenesis of liver damage in patients with chronic liver cholestasis^47,48^. In addition, PGC-1α stimulates MFN-2 mRNA and protein expression under altered energy-expenditure conditions, such as cold exposure, fasting, and IR, to control mitochondrial energy metabolism in the skeletal muscle, heart, and liver^49–51^. Our results show that nobiletin decreased DRP-1 and increased MFN-2 and OPA-1 expression to protect the mitochondria from excessive fragmentation and functional failure during hepatic IR injury.

Autophagy is activated when cells are under metabolic, ischemic, or hypoxic stress. Enhancing autophagy improves liver function by eliminating the abnormal or dysfunctional mitochondria^52^. Inhibiting autophagy increases mitochondrial oxidative stress and accelerates apoptotic and necrotic cell death during hepatic IR injury^53^. Thus, autophagy induction could restore mitochondrial function after acute liver damage^54^. A previous study showed that nobiletin attenuates acute myocardial infarction by restoring impaired autophagic flux^22^. In this study, we revealed the molecular mechanism of autophagy induction by nobiletin during hepatic IR.

The FOXO family regulates autophagy in various organs, including the skeletal muscle, cardiomyocytes, and liver^31,55^. In addition, ethanol treatment increases the expression of autophagy-related genes through FOXO3a activation in mouse liver and primary hepatocytes^56^. FOXO3a activity is regulated by posttranslational modifications, including phosphorylation, acetylation, and ubiquitination^16^. Natural compound (dihydromyricetin or berberine) treatment reduces hepatic IR injury by enhancing the SIRT-1/FOXO3a-induced autophagy pathway; FOXO3a siRNA or SIRT-1 inhibition diminishes the protective effect of these compounds^27,57^. SIRT-1 activation also protects against pulmonary emphysema via deacetylation of FOXO-3a and reduction of premature senescence in mice^58^. Here, we found that nobiletin treatment increased SIRT-1 and FOXO3a expression, inducing autophagy during hepatic IR injury, and this protective effect against hepatic IR was abolished by SIRT-1 inhibition. These data indicate that activation of autophagy via the SIRT-1/FOXO3a pathway is crucial for nobiletin-mediated protection. However, further epigenetic studies are required to determine the mechanism of action of nobiletin regarding the regulation of SIRT-1 expression, either through direct interaction or indirectly.

FOXO3a is phosphorylated by the serine/threonine protein kinase AKT and becomes sequestered by 14–3–3 proteins in the cytoplasm, which inhibits FOXO3a transcriptional activity^28^. Ethanol treatment decreases the levels of phosphorylated AKT and FOXO3a, which in turn increases FOXO3-mediated transcription of autophagy-related genes. However, deletion of the farnesoid X receptor impairs FOXO3a-mediated autophagy and exacerbates alcohol-induced liver injury. Resveratrol, a SIRT-1 agonist, enhances ethanol-induced expression of autophagy-related genes through FOXO3a deacetylation^30,56^. Here, nobiletin increased SIRT-1 and FOXO3a expression and FOXO3a nuclear translocation, simultaneously reducing phosphorylation of AKT and FOXO3a in the cytosol and resulting in FOXO3a-mediated autophagy induction. We hypothesize that nobiletin attenuates IR-induced liver damage by enhancing autophagy through the PI3K/AKT/FOXO3a and SIRT-1/FOXO3a pathways.

Our results indicate that nobiletin may contribute to epigenetic control of the SIRT-1 promoter and/or trans-acting factors to upregulate SIRT-1 expression. Similarly, resveratrol, a specific SIRT-1 agonist, has been shown to upregulate SIRT-1 expression and protect against cerebral IR injury, radiation-induced intestinal injury, and sepsis-induced myocardial injury^59–61^. In addition to natural polyphenols such as resveratrol, recent studies have identified small molecule SIRT-1 activators with enhanced potency, such as imidazothiazoles (e.g., SRT1720) and molecules bearing benzimidazole and urea-based scaffolds^62^. These SIRT-1 activators enhance the substrate affinity through an allosteric mechanism, particularly through a conserved glutamate (E230) in the *N*-terminal activation domain of SIRT-1, and facilitate formation or stabilization of the activated SIRT-1 conformation^63^. Apparently, the structure of nobiletin is not consistent with these identified SIRT-1 activators, and the crystal structure of the nobiletin–enzyme complex has not yet been revealed, unlike resveratrol or other SIRT-1 activators^64,65^. We speculate that nobiletin may stabilize an enzyme–substrate interaction to activate SIRT-1 in a FOXO3a substrate-specific manner by strengthening the binding of “loose-binding” substrates, similar to the action of resveratrol on p53^66^. Since the effect of nobiletin was abolished by EX-527, nobiletin potentially acts by enhancing SIRT-1 enzymatic activity; however, further characterization is required to determine the specific molecular mechanisms of nobiletin.

Nobiletin is metabolized by cytochrome P450 enzymes, and the resulting metabolites exert different biological and pharmacological activities^67^. Nobiletin is a promising candidate for drug development, but the target receptors need to be verified so that nobiletin can be used precisely for clinical applications. Recent studies have identified several metabolites of nobiletin with biological activities, including 3′,4′-didemethylnobiletin, 5-demethylnobiletin, and 4′-demethylnobiletin. These metabolites exhibit significant and often greater effects than nobiletin with respect to the reduction of inflammation, oxidative stress, carcinogenesis, and atherogenesis^33^. The nobiletin metabolites that enhance autophagy and/or mitochondrial function have yet to be studied; however, the aforementioned metabolites could be promising candidates and could facilitate the identification of the specific target receptors of nobiletin.

In summary, this study is the first to demonstrate that nobiletin protects against hepatic IR injury through the induction of autophagy and homeostatic control of mitochondrial biogenesis and dynamics. These effects occur through SIRT-1/FOXO3a-mediated autophagy and SIRT-1/PGC-1α-mediated mitochondrial regulation.

## Supplementary information

Supplementary Figures
