# Supplementary material for: Nobiletin ameliorates hepatic ischemia and reperfusion injury through the activation of SIRT-1/FOXO3a-mediated autophagy and mitochondrial biogenesis
Source: Exp Mol Med. 2019 Apr 26;51(4):51. doi: 10.1038/s12276-019-0245-z (PMC6486618; doi:10.1038/s12276-019-0245-z)
Supplement: Supplementary file 1 — Supplementary Figures [file 12276_2019_245_MOESM1_ESM.docx]

**
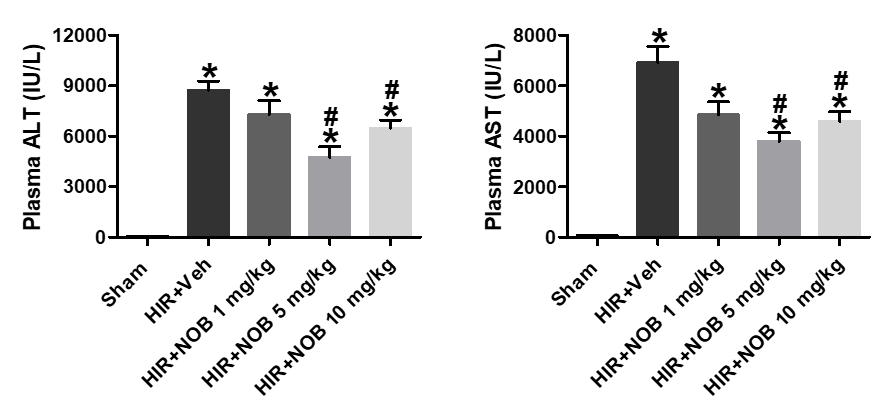
**

**Supplementary figure 1.** Plasma ALT and AST levels. Mouse livers were subjected to ischemia of 60 min and followed by 5 h of reperfusion and nobiletin was treated with concentrations of 1, 5 or 10 mg/kg at the start of reperfusion. The data are presented as mean ± SEM (n=6), **p*<0.05 versus Sham group; **^#^***p*<0.05 versus HIR+Veh group.


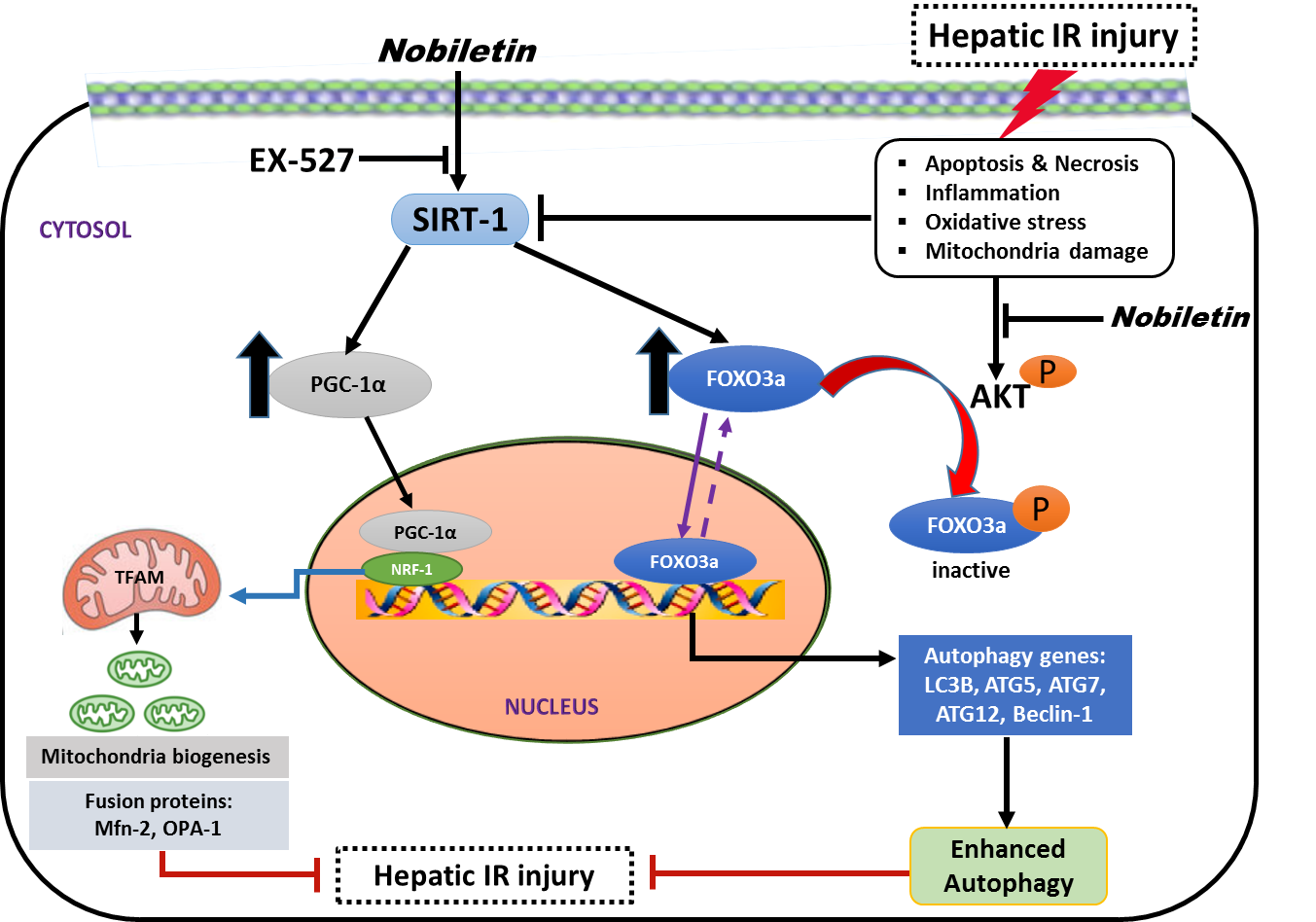


**Supplementary figure 2.** Schematic molecular mechanism of nobiletin-mediated protection during hepatic IR injury. SIRT-1 is an essential upstream regulator of PGC-1α and FOXO3a, enhancing mitochondrial and autophagy function, and protecting the liver against hepatic IR injury.
